# Listeriolysin O Is Strongly Immunogenic Independently of Its Cytotoxic Activity

**DOI:** 10.1371/journal.pone.0032310

**Published:** 2012-03-05

**Authors:** Javier A. Carrero, Hector Vivanco-Cid, Emil R. Unanue

**Affiliations:** 1 Department of Pathology and Immunology, Washington University School of Medicine, St. Louis, Missouri, United States of America; 2 Instituto de Investigaciones Médico Biológicas, Universidad Veracruzana, Veracruz, Mexico; University of Bergen, Norway

## Abstract

The presentation of microbial protein antigens by Major Histocompatibility Complex (MHC) molecules is essential for the development of acquired immunity to infections. However, most biochemical studies of antigen processing and presentation deal with a few relatively inert non-microbial model antigens. The bacterial pore-forming toxin listeriolysin O (LLO) is paradoxical in that it is cytotoxic at nanomolar concentrations as well as being the source of dominant CD4 and CD8 T cell epitopes following infection with *Listeria monocytogenes*. Here, we examined the relationship of LLO toxicity to its antigenicity and immunogenicity. LLO offered to antigen presenting cells (APC) as a soluble protein, was presented to CD4 T cells at picomolar to femtomolar concentrations- doses 3000–7000-fold lower than free peptide. This presentation required a dose of LLO below the cytotoxic level. Mutations of two key tryptophan residues reduced LLO toxicity by 10–100-fold but had no effect on its presentation to CD4 T cells. Thus there was a clear dissociation between the cytotoxic properties of LLO and its very high antigenicity. Presentation of LLO to CD8 T cells was not as robust as that seen in CD4 T cells, but still occurred in the nanomolar range. APC rapidly bound and internalized LLO, then disrupted endosomal compartments within 4 hours of treatment, allowing endosomal contents to access the cytosol. LLO was also immunogenic after *in vivo* administration into mice. Our results demonstrate the strength of LLO as an immunogen to both CD4 and CD8 T cells.

## Introduction

LLO is an immunological enigma: it is both a major virulence determinant and a major immunogen following *L. monocytogenes* infection, yet it is a highly cytotoxic protein. LLO, a member of the cholesterol-dependent cytolysin (CDC) family of bacterial toxins [1], [2], is a pore-forming protein, capable of lysing red blood cells, and inducing necrotic, pyroptotic, and apoptotic forms of cell death in nucleated cells [3]–[8]. Intracellular *L. monocytogenes* requires LLO to escape the phagosome and survive in infected cells. LLO-deficient *L. monocytogenes* (Δ*hly*) are not pathogenic and are poorly immunogenic even at high doses [9]–[12]. LLO production is carefully controlled by the microbe, through both transcriptional and post-translational mechanisms to prevent the early destruction of the infected cell [13]–[15]. *L. monocytogenes* engineered to have uncontrolled LLO activity are less virulent because they destroy their protective host niche [16].

During infection with *L. monocytogenes* a CD4 and CD8 T cell response is directed to LLO [9], [17]–[20]. Moreover, DNA vaccines containing LLO sequences fused to tumor-associated antigen induce specific immune responses to tumors [21]. In brief, LLO is immunogenic when presented as part of microbes or DNA vaccines. Yet, in vivo as well as in culture assays, LLO is a strong apoptogenic protein that causes the death of T cells as they became activated, thereby inhibiting their responses [22]. Additionally, purified LLO causes cell death in dendritic cells (DC) [7]. LLO tested as a purified protein in culture assays is poorly immunogenic causing marked negative effects on cells [17], [23].

In this work, we attempt to explain these apparently contradictory issues regarding LLO antigenicity and immunogenicity focusing on its effects on APC and the T cell response. We found LLO to be one of the strongest generators of CD4 T cell responses we have tested. It elicited CD4 T cell responses at unprecedented [fM]/[pM] levels, approximately 3000–7000 times more efficiently than the responses to the cognate peptide. LLO was also presented to CD8 T cells. Importantly, the immunogenic and cytotoxic activities of LLO were distinct because LLO mutants having much reduced cytotoxicity were processed and presented equivalently to the wild-type protein and elicited *in vivo* immune responses. The parameters of LLO binding, uptake, and catabolism by APC, as well as breakdown of endosomal vesicles were included in this evaluation.

## Results

### Cytotoxicity of LLO

**Figure 1:**
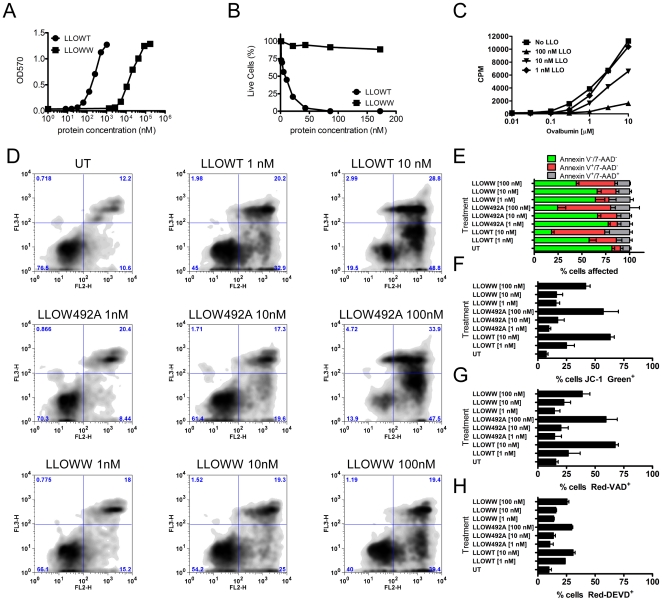
Reduced toxicity of LLO tryptophan mutants. **A**. LLOWW was tested for hemolytic activity on human red blood cells as in [46]. LLO was added at the indicated concentrations for 60 min. at 37°C then, hemoglobin release was measured at OD570. **B**. C3.F6 [52] cell line was incubated with the indicated concentrations of LLOWT or LLOWW for 1 hr. at 37°C. Cells were stained with trypan blue and percentage of live cells (trypan negative) was determined. **C**. An ovalbumin-responsive primary T cell line (5×10^4^) [6] was incubated for 16 hr. with BMDC (5×10^4^) and the indicated concentrations of ovalbumin (antigen) and LLO (toxin). T cell responses were measured by IL-2 production. **D-H**. BMDC (1×10^6^) were treated with the indicated LLO variants at the given concentrations for 6 hours. Cell death was measured by (**D–E**) Annexin V/7-AAD staining, (**F**) JC-1 dye, (**G**) Red-VAD, or (**H**) Red-DEVD. All cell death analyses were performed by flow cytometry. Cells were gated for CD11c^+^ events then the percentage of affected CD11c^+^ cells was plotted. Results in (**A–C**) are representative of two independent experiments. Bars in (**D–H–F**) represent the mean±S.D. of at least 2 independent experiments performed in duplicate or triplicate (n = 5–7).

We examined the antigenicity of soluble LLO (referred to as LLOWT) as well as of LLO having two key tryptophans mutagenized to alanines [24]. These residues are part of a highly conserved undecapeptide sequence (483-ECTGLAWEWWR-493) involved in the pre-pore to pore transition when CDC family members bind to membranes [25]. Mutation of the tryptophans to alanines at both residues 491 and 492 (LLOWW), or at only residue 492 (LLO492A) led to a reduction in hemolytic activity of ∼95–99.5% (Fig. 1A) and in cytolytic activity to nucleated cells (Fig. 1B). Also, LLO inhibited responses of primary T cells in a dose dependent manner (Fig. 1C).

The concentration of LLO required to induce cell death in DCs and macrophages was examined. Bone marrow-derived DCs (BMDC) (Fig. 1D–H) and bone marrow-derived macrophages (BMM) (not shown) were treated with different concentrations of LLOWT, LLOW492A, or LLOWW for 6 hours, and cell death was measured using Annexin V/7-AAD (Fig. 1D–E), JC-1 (Fig. 1F), Red-VAD (pan-caspase; Fig. 1G), and Red-DEVD (caspase-3; Fig. 1H) staining. All four assays showed apoptosis of BMDC and BMM at ∼1 nM of LLOWT while the LLOW492A and LLOWW induced apoptosis between 10 and 100 nM. The dose of LLOWT required for cell death coincided with the drop in the T cell response detected in our T cell assays (see below).

**Figure 2:**
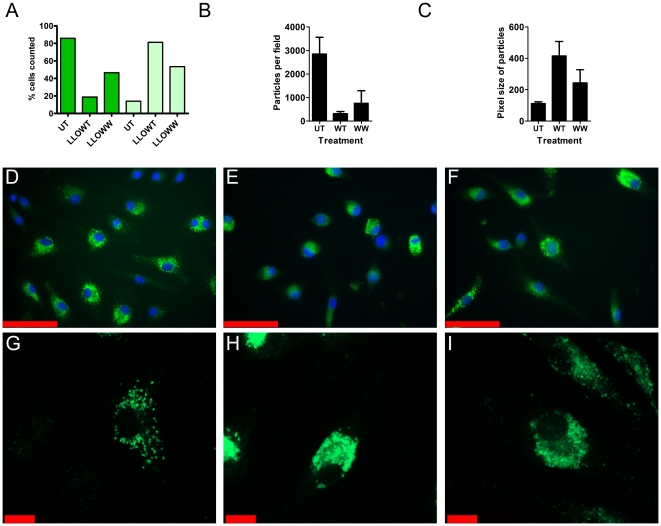
Release of vesicular dextrans into the cytosol. **A–I.** BMM (1.25×10^5^) were adhered to 12 mm coverslips and treated with FITC-dextran for 30 min then either left untreated (**D,G**) or treated with LLOWT (**E,H**) or LLOWW (**F,I**) for 4 hours. **A.** The number of cells containing punctate staining was determined by counting ∼100 individual cells for each treatment. A cell was considered to have puncta if it had any visible discrete spots, as in panel **G**. Cells were considered to have no puncta if the green staining was homogeneous, as in panel **H**. **B–C.** ImageJ analysis of the number of particles and size of particles per field was determined as detailed in the materials and methods. Bars represent the mean+/−S.D. particles per field (**B**) or pixel size of particles (**C**) for three independent fields with 10–24 cells per field. **D–F.** Representative epiluminescence images taken following treatment with FITC-Dextran and then either left untreated (**D**) or treated with 1 nM LLOWT (**E**) or 10 nM LLOWW (**F**). The nucleus (DAPI) was false colored blue and FITC-Dextran was false colored green. **G–H.** Representative laser scanning micrographs of FITC-dextran loaded BMM either untreated (**G**) or treated with 1 nM LLOWT (**H**) or 10 nM LLOWW (**I**). Scale bars in represent 50 µm (**D–F**) or 10 µm (**G–I**). Microscopy is representative of 2 independent experiments performed in duplicate.

The mechanisms of cell death induced by LLO are not entirely understood. LLO is an endosomolytic agent that can cause cell death from within a cell by releasing intracellular stores of granzymes [6], [26]. Based on this information APC were examined for release of intracellular endosomal contents. Fluorescein-labeled low molecular weight dextrans (3 kDa) were given to BMM, and then examined with or without LLO treatment. Untreated cells displayed a mostly punctate green staining corresponding to previously reported endosomal localization of the dextrans (Fig. 2A, D, G) [27]. Approximately 80% of dextran-labeled untreated cells contained punctate vesicular staining with no diffuse cytosolic staining (Fig. 2A). ImageJ was used to count both the number of particles (puncta) per field and the pixel size of the particles (Fig. 2B–C). Following 4 hr. of LLOWT treatment, cellular morphology was altered, and ∼20% of the cells had a punctate staining, while ∼80% showed the diffuse patterns shown in Fig. 2E, H. We also detected an increase in the size of FITC-Dextran+ compartments present in LLOWT treated cells (Fig. 2C). LLOWW did not cause an obvious change in the cellular morphology, but still led to ∼60% of the cells possessing a cytosolic distribution of dextran and reduced number of puncta as well as increased particle size (Fig. 2B–C, F, I). These data show that LLO releases endosomal contents into the cytosol, providing a possible mechanism for entry of endocytosed antigens into the class I MHC processing pathway, discussed below.

### Processing and presentation of LLOWT

The experiments only examine the processing and presentation of LLO independent of the costimulatory activity of the APC. For this, T cell hybridomas were used since their responses are quantitatively related to the amount of peptide-MHC complexes presented by the APC. CD4 T cell hybridomas were generated against the 190–201 segment of LLO [18] and tested against the various LLO preparations.

**Figure 3:**
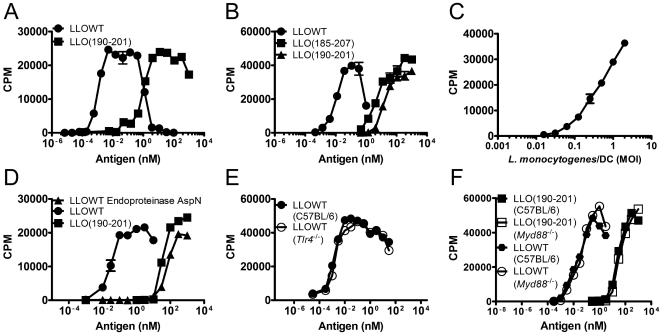
Enhanced presentation of an LLO protein-derived CD4 T cell epitope. **A–B.** 5×10^4^ I-A^b^-restricted LLO(190–201)-specific hybridomas 57-3 (**A**) or LL73 (**B**) were incubated for 16 h with 10^5^ PEC (**A**) or 5×10^4^ BMDC (**B**) from C57BL/6 mice and various concentrations of either LLOWT, LLO(190–201), or LLO(187–205). **C.** BMDC (10^5^) were infected with the indicated MOI of *L. monocytogenes* EGD strain as in [45] and 16 hrs. later the response of 57-3 (5×10^4^) was determined. **D.** LLO was treated with endoproteinase AspN or buffer for ∼16 hr. at 37°C then incubated with for ∼16 hr. with 10^5^ irradiated C57BL/6 splenocytes and 57-3 (5×10^4^). *E*, *Tlr4^−/−^*, (**F**) *Myd88^−/−^* (KO), or (**E–F**) C57BL/6J (WT) irradiated splenocytes (10^5^) were treated with LLOWT or LLO(190–201) and (**E**) 57-11 or (**F**) 57-3 T cell hybridomas (5×10^4^). Following all incubations, T cell hybridoma activation was determined by CTLL-2 assay. Points indicate the mean±S.D.

In any given T cell assay, LLOWT was presented to CD4 T cells at low picomolar to high femtomolar concentrations: the presentation was ∼1000–10000-fold more efficiently than the presentation of the peptide LLO(190–201) (Fig. 3A). There was a ∼20-fold improvement in presentation of LLO(185–207) over LLO(190–201), but this effect was not responsible for the improved presentation of LLOWT. The enhanced presentation was found with a variety of APC, including peritoneal exudate macrophages (PEC) (Fig. 3A), BMDC (Fig. 3B), BMM, irradiated splenocytes, and purified splenic CD11b^+^ and CD11c^+^ cells (data not shown). The response of T cell hybridomas was initially detected at the 0.1–10 pM range, peaked between 10–100 pM, and dropped at 1–10 nM. The drop corresponded to the doses that induced cytolytic and apoptotic effects on APC and T cells (Fig. 1) [22]. The LLO-reactive CD4 T cell hybridomas also responded to DC infected with live *L. monocytogenes* at multiplicities of infection (MOI) ranging from 0.1–5 (Fig. 3C). The enhanced presentation of LLOWT required intact tertiary structure because digestion with endoproteinase AspN yielded the LLO(164–204) fragment that was presented at equivalent doses to the LLO(190–201) peptide (Fig. 3D). LLO preparations were purified with polymyxin B columns to remove LPS and yielded the same enhanced antigenicity (data not shown). *Tlr4* ^−/−^ and *Myd88* ^−/−^ splenocytes presented LLOWT equivalently to wild-type counterparts (Fig. 3E–F), further demonstrating that LPS contamination was not responsible for the enhanced presentation of LLO. Treatment of LLOWT with cholesterol had no effect on its presentation to hybridomas (data not shown).

### Processing and presentation of LLO is independent of its immediate cytotoxicity

**Figure 4:**
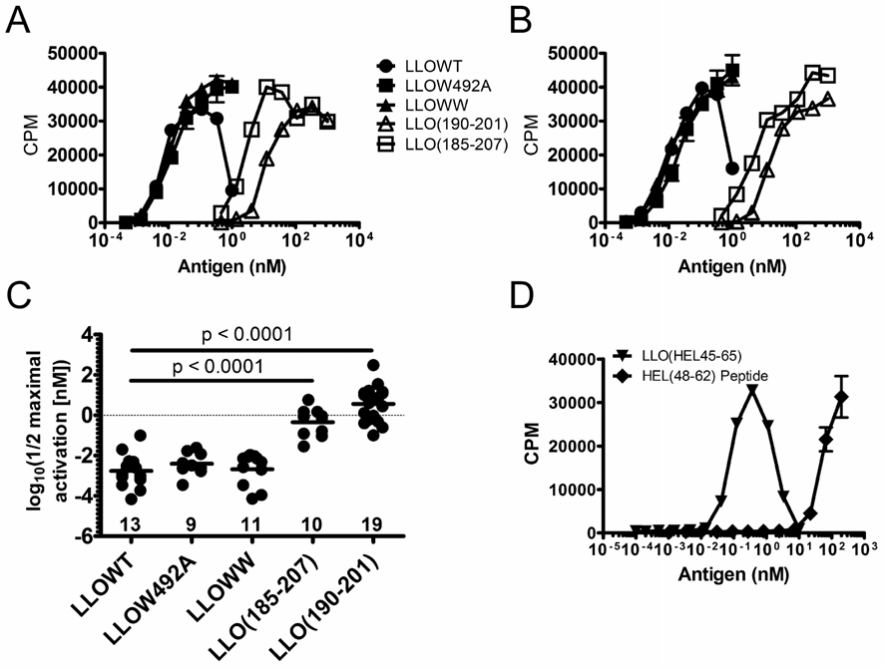
Antigenicity of LLO to CD4 T cells is independent of cytotoxicity. **A–B.** C57BL/6 BMDC (5×10^4^) and the LLO-specific hybridomas (5×10^4^) (**A**) 57-3 or (**B**) LL73 were incubated with the indicated concentrations of LLO variants or the peptides for ∼16 hrs and T cell activation was determined. **C.** The half-maximal dose of antigen required to activate T cell hybridomas in 62 independent assays was calculated using non-linear regression fitting of the dose-response plots. The plot shows the log_10_ of the computed half-maximal activation value. Each dot represents and individual T cell assay. The geometric mean is given for each column. p values were calculated by student T test following validation of normal distribution through D'Agostino & Pearson omnibus normality test. There was a statistical significance in the reduced dose requirement for presentation of LLOWT, LLOW492A, and LLOWW when compared to LLO(190–201) or LLO(185–207) peptides (p<0.0001). **D.** B10.BR PEC (10^5^) and the 3A9 T cell hybridoma specific for HEL(48–62) (5×10^4^) were incubated with either HEL(48–62) peptide or LLO(HEL48–65) fusion protein for ∼16 hr. and tested for T cell activation by CTLL-2 assay. For (**A**), (**B**), and (**D**) dots represent the mean±S.D. for triplicate values at each antigen dose.

LLOW492A and LLOWW were presented by either BMDC (Fig. 4A–B) or BMM (not shown) at the same concentrations as LLOWT despite requiring higher doses to induce cell death.

Fig. 4C shows the summary of 62 independent T cell assays performed with 4 different hybridomas and either BMDC or BMM as the APC. The mean half-maximal activation dose was not significantly different between the three LLO protein preparations: for LLOWT, LLOW492A, LLOWW, 3.3 pM, 7.5 pM, 4.7 pM respectively, compared to 1.2 nM, and 23 nM for LLO(185–207) and LLO(190–201), respectively. These amounted to an average fold-increase of presentation for LLOWT, LLOW492A, LLOWW, and LLO(185–207) of ∼7000, ∼3000, ∼5000, and ∼20-fold respectively when compared to LLO(190–201). Importantly, the doses of all three LLO variants required to reach half-maximal activation of T cells were >1000-fold lower than the doses required to induce cell death (∼1–10 pM versus ∼1–10 nM).

We wanted to determine if LLO protein could enhance the presentation of an ectopic antigenic specificity. A construct was generated that contained the 45–65 segment of the hen-egg white lysozyme (HEL) protein fused to the amino terminus of LLO. There was a ∼1000-fold enhancement in presentation of LLO-HEL(45–65) compared to presentation of the HEL(48–62) peptide (Fig. 4D). The response of 3A9 T cells to the HEL(48–62) peptide and to HEL protein was identical [28]. The enhancement of presentation required that the peptide be covalently fused to LLO because treatment of cells with LLO and free HEL(48–62) peptide did not affect the presentation of the peptide (data not shown).

### Quantification of the uptake, binding and catabolism of LLO

**Figure 5:**
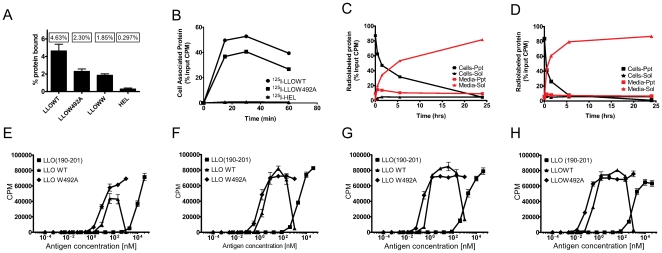
Binding, uptake and catabolism of radiolabeled LLO. **A.** BMM (10^6^) were treated with ∼150 ng of ^125^I-LLO or HEL for 30 min. on ice. The amount of CPM bound to cells or found in the supernatant was measured and a percent of total bound to cells was calculated. **B.** ^125^I-LLO, LLOW492A, or HEL (∼50 ng) were given to BMM for 15, 30, or 60 min. and the amount of incorporation of radioactivity was measured. Values are plotted as a percent of total input radioactivity that was taken by the cells. **C–D.** BMM (10^6^) were treated with 50 ng ^125^I labeled LLOWT (**C**) or LLOW492A (**D**) for the indicated times. Then, TCA precipitation was performed and the amount of total radioactivity present in the precipitate (ppt) or supernatant (sol) was measured. The ppt fraction contains large polypeptides and represents the non-catabolized fraction and the sol fraction contains small peptides and free amino acids and represents the catabolized fraction. Similar uptake and catabolism results were obtained with BMDC. Each experiment in (**A–D**) is representative of at least two independent experiments performed in triplicate. **E–F.** BMM (10^5^) were incubated with LLO(190–201 peptide), LLOWT, or LLOW492A at the indicated antigen concentrations for 15 (**E**), 30 (**F**), 60 (**G**) or 120 (**H**) min. BMM were fixed with PFA, quenched with lysine, washed and then LLO specific 57-3 T cell hybridoma (5×10^4^) was added for ∼16 hr. T cell activation was determined by CTLL-2 assay. Experiment is representative of 2 independent experiments performed in triplicate. Similar results were obtained with BMDC.

The uptake and catabolism of LLO was examined in order to relate it to its antigenicity. LLO was labeled with ^125^Iodine and its binding, uptake, and catabolism by APC was evaluated. Radiolabeling did not affect the hemolytic activity of LLOWT, LLOW492A, or LLOWW (data not shown). All LLO preparations bound to BMM incubated on ice, at the levels shown in Figure 5A, ranging from 2–4% of input cpm. In contrast the proteins HEL-^125^I bound to BMM at ∼0.297% of input. As noted the mutation of tryptophans reduced, but did not prevent LLO from binding to cells. LLO-^125^I was internalized rapidly by BMM (Fig. 5B) and BMDC (data not shown) following incubation at 37°C. About 40–50% of total input LLO was found as cell-associated protein within 30 minutes. There was ∼15–20% reduction in total internalization of LLOW492A (Fig. 5B) or LLOWW (not shown) compared to LLOWT. In contrast, HEL-^125^I was internalized at ∼0.8% of total input protein. LLOWT was rapidly catabolized, with ∼20% of total ^125^I counts found in the TCA soluble fraction of the cell culture media within 1 hour of treatment (Fig. 5C). By 24 hr., >80% of total ^125^I counts were found in the soluble fraction. Similar rates of catabolism were detected with LLOW492A (Fig. 5D) and LLOWW (not shown).

BMM and BMDC were fixed at different times following treatment with LLO to determine the minimal time required to process and present LLO. By T cell assay, presentation of LLOWT and LLOW492A by BMM was found as early as 15 min post-uptake (Fig. 5E). The enhancement in presentation was already detectable by 15 min, became more pronounced by 30 min (Fig. 5F), and peaked between 60 min (Fig. 5G) and 120 min post-treatment (Fig. 5H). Similar results were obtained with BMDC (not shown). Therefore, by two independent measures, LLO was rapidly internalized, processed and presented on the surface of APC.

### Processing and presentation of LLO to CD8 T cells

**Figure 6:**
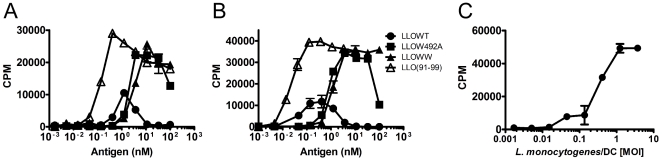
Antigenicity of LLO to a CD8 T cell was not dependent on cytotoxicity. **A–B.** H-2K^d^-restricted LLO(91–99)-specific hybridoma 206.15 (5×10^4^) were incubated for ∼16 hr. with 10^5^ PEC (**A**) or 5×10^4^ BMDC (**B**) from BALB/c mice and various concentrations of either LLOWT, LLOW492A, LLOWW, or LLO(91–99). **C.** 206.15 (5×10^4^) was incubated with the BMDC (10^5^) infected at the indicated MOI of *L. monocytogenes* EGD strain [45]. Following all incubations, T cell hybridoma activation was determined by CTLL-2 assay. All T cell activation assays are representative of at least two independent experiments performed in triplicate. Points indicate the mean±S.D.

LLO provides epitopes for presentation on the class I-MHC pathway following *in vivo* infection with *L. monocytogenes* [29]. We tested if the LLO could be processed and presented to CD8 T cells *in vitro*. For these experiments, the LLO(91–99) peptide presented by the class I MHC protein H-2K^d^ was examined [19]. Presentation of LLOWT induced weak activation of the LLO(91–99)-responsive CD8 T cell hybridoma (Fig. 6A–B). In contrast presentation of LLOW492A and LLOWW was stronger and the half-maximal response dose was higher than for the LLO(91–99) peptide (Fig. 6A–B). It was not surprising that LLOWT was not well presented because the doses required to elicit CD8 T cell responses to LLOW492A and LLOWW was in the range where LLOWT caused cytolysis and apoptosis of nucleated cells (∼1–10 nM, Figs. 1, 3, and 4). CD8 T cell activation was elicited with as little as ∼1 nM LLO presented by macrophage cell lines (not shown) and ∼10 nM LLO presented by PEC and BMDC (Fig. 6A–B). CD8 T cell hybridomas against LLO also responded to *L. monocytogenes*-infected DC at MOI ranging from 0.1 to 5 (Fig. 6C).

### *In vivo* response to LLO immunization

**Figure 7:**
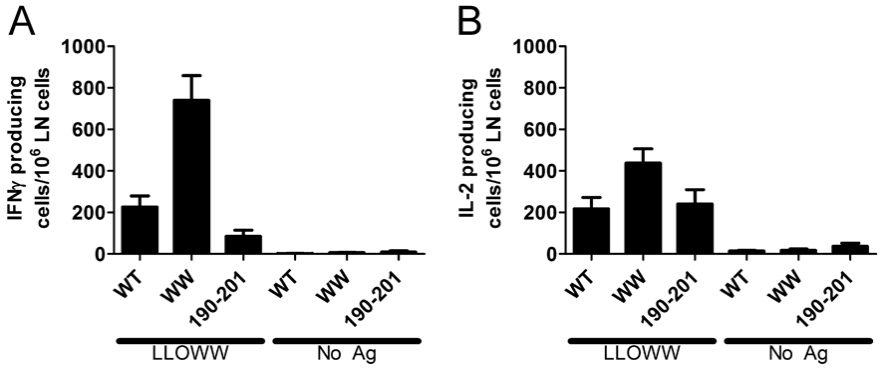
ELISpot response following immunization with LLO or LLO peptide. **A–B.** C57BL/6 mice were immunized in the footpad with 250 picomoles of either LLOWT, LLOWW, or LLO(190–201) peptide emulsified in incomplete Freund's adjuvant. After 7 days, draining lymph nodes were isolated and the number of IFNγ (**A**) and IL-2 (**B**) producing cells was determined by ELISpot. Recall antigen was either LLOWW at 10 nM or no antigen. Bars represent the mean+/−SEM for 2–6 mice per group over three independent experiments. Analysis of the data using D'Agostino & Pearson omnibus normality test followed by student t test revealed that the increase in IFNγ production following immunization with LLOWW was significant when compared to both LLOWT or LLO(190–201) immunization (p = 0.0012 for LLOWT vs. LLOWW and p<0.0001 for LLOWW vs. LLO(190–201).

LLOWT, LLOWW or LLO(190–201) were emulsified in incomplete Freund's adjuvant (oil∶water emulsion) and injected into the footpads of C57BL/6 mice. An IFNγ and IL-2 was elicited with 250 picomoles per injection from both protein and peptide immunizations (Fig. 7). The average number of spots per million lymph node cells that produced IFNγ was 226, 740, and 84 for LLOWT, LLOWW and LLO(190–201) (Fig. 7A). In the case of IL-2, the averages were 216, 438, and 240, for LLOWT, LLOWW and LLO(190–201), respectively (Fig. 7B). These responses were higher than those obtained with regular protein antigens (ovalbumin and hen-egg lysozyme).

## Discussion

We have shown that purified recombinant LLO was processed and presented to CD4 T cell hybridomas at picomolar antigen concentrations. The ability of LLO to be presented to CD4 T cells at such low concentrations was not a peculiarity of the LLO peptides that were selected for processing. Fusion of an HEL(45–65) peptide to the amino terminus of the LLO protein led to similar enhancement in presentation. We also found presentation of LLO to CD8 T cell hybridomas, albeit at nanomolar levels. The enhanced presentation of LLO was independent of its cytotoxic qualities. Mutants of LLO that caused reduced cytolysis and apoptosis had equivalent enhancement in presentation to the wild-type protein.

The relationship between immunogenicity and cytolysis explains previous studies in which LLO was shown to be an ineffective immunogen or an inhibitor of T cell activation [17], [23], [30]–[31]. At micromolar concentration, LLO leads to immediate cytolysis. But LLO can also induce apoptosis of lymphocytes and APC at high nanomolar concentrations [22]. It can also cause T cell unresponsiveness to other model antigens through an undefined effect on both APC and T cells [30]–[31]. Therefore, the amount of LLO that needs to be provided to T cell assays needs to be carefully titrated over a wide range to get the most meaningful result. Our experimental approach has been the first one to consider these parameters in examining LLO as an antigen.

LLO was internalized very rapidly by APC. Likewise, processing and catabolism was rapid, presentation occurred by 15 min after uptake, with maximal presentation at about 2 hrs. These early times indicate presentation by class II-MHC molecules before there was any signs of cytotoxicity or escape of contents into the cytosol. After 2 hours, and peaking at 4 hours of LLO treatment, there was release of vesicular dextran into the cytosol of cells. The detection of cytosolic dextran coincided with the earliest markers of apoptosis that were detectable on BMM or BMDC. Peak BMM/BMDC apoptosis occurred at ∼6 hours post-LLO treatment. Catabolic products of LLO were detected within 30 minutes of treatment, and LLO was maximally degraded by ∼6 hours of treatment.

We hypothesize that three of LLO's important biological activities: 1) high affinity membrane binding, 2) pH dependence, and 3) rapid catabolism contribute to its antigenicity. The immunogenicity of LLO to both CD4 and CD8 T cells can be maintained despite mutations that reduce the pre-pore to pore transition, and by extension toxicity (LLOW492A and LLOWW). There are residues of CDCs/LLO responsible for high-affinity cholesterol binding (Loop containing regions (LC) 1–3) [32], [33]. It is possible that altering LLOs avidity to cholesterol (by mutating LC regions) could reduce CD4 or CD8 presentation by either decreasing entry into cells or altering subcellular localization. Altering the pH dependence of LLO (using mutations such as L461T) might allow LLO to lyse endocytic vesicles before they acidify [34]. This might force LLO to become cytosolic and not enter the late lysosomal compartments, thereby reducing its presentation to CD4 without affecting CD8. Alternatively, removing the pH sensor for LLO may make it non-immunogenic because it would be immediately toxic at the cell surface and never get internalized. Finally, the amino terminus PEST-like sequence of LLO has been reported to undergo multiple post-translational modifications [13], [16], [35]. These modifications somehow regulate LLO stability and catabolism in the cytosol. Alterations in the PEST-like sequence may reduce the availability of LLO peptides to the MHC-I pathway. Ultimately, it will be interesting to determine if adding individual elements of LLO, such as the PEST-like sequence, LC-region, or the undecapeptide platform, allows for targeting of ectopic antigens to specific processing and presentations pathways.

The interactions of LLO with plasma membrane proteins are not entirely known. We have not excluded the possibility of receptor-mediated entry. Although LLO has been shown to trigger TLR4 signaling, binding affinity and direct interaction between LLO and TLR4 has not been determined [36]. Our results using *Tlr4^−/−^* and *Myd88^−/−^* cells indicate that the TLR pathway did not play a role in the enhanced antigenicity of LLO *in vitro*. Another member of the CDC family, intermedilysin, has been shown to bind the complement regulatory protein CD59 [37], demonstrating that receptor binding by a CDC is a possibility. The CDCs belong to a conserved superfamily of proteins that includes the complement and perforin proteins, all of which are highly regulated at the cell surface [38]. Although LLO does not bind to CD59, it is possible that it binds other receptors involved in complement regulation, which could accelerate its internalization and/or colocalization with class II-MHC bearing vesicles compartments.

An issue with CDC field is their transition from a membrane-associated CDC to the formation of a functional pore. Based on our current understanding, CDCs should bind to membranes loosely, form a pre-pore complex, and at sufficient CDC density form the final functional pore [39]. The antigenicity of LLO was found at concentration levels sufficient for pre-pore formation but at levels that preceded the fully functional pore. Our mutants in the tryptophan platform support this conclusion since they should be unable to form functional pores on the surface of cells [40]. Taken together, these data suggest that there is a functional domain(s) of LLO distinct from its pore-forming activity that enhances antigenicity. We are currently investigating this possibility.

Finally, our data indicate that LLO potency as an antigen did not depend on intrinsic properties of the peptide segment being presented rather than on its handling by the APC: a peptide attached to it was highly antigenic indicating that LLO is a potent delivery agent for ectopic proteins. LLO-fusion proteins have been tested as DNA vaccines, as chemical conjugates with ectopic antigens, or as antigens carried by recombinant *L. monocytogenes* [41]–[43]. These systems have been used to immunize in different ways to protect from tumor challenge. However, the peculiar affinity and trafficking of CDCs has not been exploited toward a better understanding of antigen processing and presentation biochemistry and cell biology.

Our findings need to be placed in the context of the natural infection in which LLO is strong donor of peptides both to the class I and class II-MHC pathway. How LLO becomes accessible to phagocytes during the natural infection needs to be evaluated. Several avenues for presentation by class II molecules are apparent: i) in the early vesicular compartments of APC, LLO is released and can be handled like the soluble LLO protein examined here; indeed LLO in vesicles can be targeted by specific antibodies indicating its availability [44]–[45]; ii) LLO could be released from dying cells, a possibility that we have linked to its lymphocyte apoptotic properties that take place *in vivo* [22], [46]. Concerning the epitopes presented by class I MHC molecules the logical explanation is that it results from the sojourn of the bacteria in the cytosol. This is substantiated by the lack of activation of CD8 responses by LLO-deficient *L. monocytogenes* [47]. In addition, cytosolic LLO is targeted for post-translational modification and rapid degradation by the host cell [13], [16], [35]. Peptides derived from this catabolism may enter the MHC I pathway through TAP-dependent ER trafficking.

## Materials and Methods

### Animals

#### Ethics Statement

These experiments were approved by the Division of Comparative Medicine (DCM) of Washington University School of Medicine (Association for Assessment & Accreditation of Laboratory Animal Care (AAALAC) accreditation number A3381-01). All experiments were performed under institutional (DCM) guidelines and all efforts were made to minimize suffering. The institutional (DCM) approval number for these studies was protocol number 20110150.

### Mutagenesis and purification of LLO

The pET29b expression vector containing the LLO sequence was kindly provided by Dr. Daniel Portnoy (University of California, Berkeley, CA) [34]. Site-directed mutagenesis was performed using Quickchange Site-Directed Mutagenesis kit (Agilent Technologies, Santa Clara, CA) following the manufacturer's protocol. LLO-HEL fusion proteins were generated by adding the sequence for nucleotide sequence encoding HEL(45–65) to the 5′ end of the LLOWT cloned in pET29b using PCR. Mutagenesis was confirmed by sequencing of constructs at the Washington University Nucleic Acids Core facility. All LLO variants were purified as described previously [22], [34]. For some experiments LLO was further purified by passage over Detoxy-Gel endotoxin removal columns (Thermo Fisher Scientific, Waltham, MA). Endotoxin levels were determined by chromogenic Limulus Amebocyte Lysate test following the manufacturer's instructions (Lonza, Basel, Switzerland). Endotoxin levels were <1 EU/mg of LLO protein. Purity was confirmed by SDS-PAGE followed by Coomassie Brilliant Blue staining. Concentration was determined using bicinchoninic acid reaction (ThermoFisher Scientific).

### Generation of T cell hybridomas against LLO(190–201) or LLO(91–99)

Mice (C57BL/6 or BALB/c) were infected intraperitoneally with 10^3^ colony-forming units of *L. monocytogenes* EGD strain. Seven days following infection, mice were sacrificed, spleens were removed, and single-cell suspensions were generated. Isolated spleen cells were cultured with LLO(190–201) or LLO(91–99) peptide for 3 days then fused to BW5147 CD4+ or CD8+ to generate stable hybridomas using a previously described protocol [48]. T cell hybridomas were cloned at one cell per well and then tested for specificity to LLO(190–201) or LLO(91–99) peptides presented by peritoneal exudate cells (PEC).

### Antigen Presentation Assays

All cell culture work was performed in DMEM (Life Technologies, Carlsbad, CA) containing 10% (v/v) fetal calf serum (Thermo Fisher Scientific), 37°C and 5% CO_2_ unless otherwise specified. BMM were prepared as described previously [49] except that L-cell conditioned media was withdrawn at the 7th day of culture and cells were used at the 10–12th day of culture. BMDC were prepared as in [50]. T cell assays were performed by combining 5×10^4^ or 10^5^ APC, 5×10^4^ T cell hybridomas, and antigen for ∼16 hr. Culture supernatants were tested for IL-2 production by CTLL-2 proliferation assay using ^3^H-thymidine incorporation. For fixation experiments, BMM (10^5^) were allowed to internalize antigen for 15–120 min., cells were washed 3 times in DMEM, fixed with 2% paraformaldehyde (Sigma-Aldrich, St. Louis, MO) in phosphate buffered saline pH 7.4 for 15 min. at 25°C, washed 3 times, then incubated with 0.2 M lysine (Sigma-Aldrich) for 30 min. at 25°C. Then, cells were washed 3 times with DMEM/10% FCS and T cell hybridomas (5×10^4^) in DMEM/10% FCS were added for ∼16 hrs. T cell activation was determined by CTLL-2 assay. All antigen presentation assays were performed in 200-µL final volume in 96-well tissue culture coated flat-bottom plates (Corning, Lowell, MA). All data plotting and statistical calculations were performed with Prism 5.0 (GraphPad Software Inc., La Jolla, CA).

To determine *in vivo* responses to LLO, mice were immunized with 250 pmol of LLOWT, LLOWW or LLO(190–201) in 1∶1 oil∶saline emulsion (incomplete Freund's adjuvant:0.9% pyrogen-free saline) in the footpads. Seven days later, mice were sacrificed and popliteal lymph nodes were isolated and dispersed. Cells were plated on cytokine capture antibody pre-coated Multiscreen-IP plates (Millipore, Billerica, MA) with or without recall antigen (LLOWW at 10 nM) for 16 hours. ELISpot assay was then used to determine the number of interferon-γ (IFNγ) and interleukin-2 (IL-2) producing cells (BD Biosciences, San Diego, CA). ELISpot assay was performed following the manufacturer's specifications (BD Biosciences). ELISpot plates were scanned and spots were enumerated using CTL-ImmunoSpot S5 Analyzer (C.T.L., Shaker Heights, OH).

### LLO and HEL radiolabeling, binding, uptake and catabolism

LLO and HEL were radiolabeled with ^125^I using the chloramine-T method [51]. For cell surface binding experiments, 4×10^5^ BMM were incubated with ∼150 ng of LLO or HEL (specific activity ∼7×10^3^ CPM/ng protein) in 200 µL of DMEM/1% FCS in 1.7 mL siliconized tubes for 30 min. on ice. Cells were spun through oil, and radioactivity was counted in the supernatant versus cellular fraction (60%/40% dibutyl phthalate/dioctyl phthalate, vol/vol; Thermo-Fisher Scientific). For uptake experiments, 2×10^6^ BMM were incubated with 50 ng LLO or HEL (specific activity ∼2–5×10^3^ CPM/ng of protein) in 1 mL DMEM/10% FCS for 15 min at 25°C. Cells were centrifuged and washed twice with DMEM/10% FCS and plated in 1 mL volume for 0–60 min. at 37°C. At different, times cells were removed and spun through oil to remove free radioactivity. For catabolism experiments, the same protocol was followed except that at the appropriate times, cells and media were harvested separately and adjusted to 10% trichloroacetic acid (TCA; Sigma-Aldrich) to precipitate protein. TCA precipitate was centrifuged for 30 min. at 10,000 RCF, and the amount of radioactivity in the pellet (proteins) and supernatant (small peptides and free amino acids) was determined. The amount of radioactivity in all fractions was determined using a gamma counter (LKB Wallac 1272 CliniGamma, Perkin Elmer Inc., Waltham, MA).

### Immunofluorescence of dextran location

For FITC-dextran treatment, 10^5^ BMM adhered to 12-mm glass coverslips were treated with 0.5 mg/mL FITC-dextran (Life Technologies) in DMEM/1% FCS for 30 minutes. Cells were then washed 3 times with DMEM/1% FCS and incubated with LLO in DMEM/1% FCS or left untreated for 4 hr. Following incubation, cells were washed with 1 mL PBS 3 times, fixed with 0.5 mL of 2% PFA in PBS, washed 3 times with 1 mL DMEM/10% FCS, washed 3 times with 1 mL PBS, then mounted to a slide with Prolong Gold. Images were acquired using either an Olympus BX51 (Olympus, Center Valley, PA) with a 100W mercury source or a Zeiss Axiovert 100M microscope with a LSM510 source (Carl Zeiss, Inc., Thornwood, NY). On the BX51, a UPlanFl 60×/1.25 objective was used. On the Axiovert 100M either a 40×/1.3 FLUAR or a Plan Apochromat 63×/1.4 objective was used. Spot Advanced (Diagnostic Instruments, Inc., Sterling Heights, MI) was used to acquire epiluminescence images on the BX51. LSM510 Version 3.2 (Carl Zeiss, Inc.) software was used to acquire/analyze images and determine colocalization of LLOWW with MHC II on the Axiovert 100M/LSM510. Quantification of pixel density and particle size was performed with ImageJ (NIH, Bethesda, MD). Images were thresholded using the default setting then the analyze particle feature was used to calculate pixel number and size of particles in multiple fields. Microscopy images were resized and adjusted for brightness and contrast using Adobe Photoshop CS4 (Adobe Systems Inc., San Jose, CA).
